# Assessment of Proton Beam Therapy Use Among Patients With Newly Diagnosed Cancer in the US, 2004-2018

**DOI:** 10.1001/jamanetworkopen.2022.9025

**Published:** 2022-04-27

**Authors:** Leticia M. Nogueira, Ahmedin Jemal, K. Robin Yabroff, Jason A. Efstathiou

**Affiliations:** 1 Department of Surveillance and Health Equity Science, American Cancer Society, Atlanta, Georgia; 2 Department of Radiation Oncology, Department of Radiation Oncology, Massachusetts General Hospital, Boston

## Abstract

**Importance:**

Proton beam therapy (PBT) is a potentially superior technology to photon radiotherapy for tumors with complex anatomy, those surrounded by sensitive tissues, and childhood cancers.

**Objective:**

To assess patterns of use of PBT according to the present American Society of Radiation Oncology (ASTRO) clinical indications in the US.

**Design, Setting, and Participants:**

Individuals newly diagnosed with cancer between 2004 and 2018 were selected from the National Cancer Database. Data analysis was performed from October 4, 2021, to February 22, 2022. ASTRO’s Model Policies (2017) were used to classify patients into group 1, for which health insurance coverage for PBT treatment is recommended, and group 2, for which coverage is recommended only if additional requirements are met.

**Main Outcomes and Measures:**

Use of PBT.

**Results:**

Of the 5 919 368 patients eligible to receive PBT included in the study, 3 206 902 were female (54.2%), and mean (SD) age at diagnosis was 62.6 (12.3) years. Use of PBT in the US increased from 0.4% in 2004 to 1.2% in 2018 (annual percent change [APC], 8.12%; *P* < .001) due to increases in group 1 from 0.4% in 2010 to 2.2% in 2018 (APC, 21.97; *P* < .001) and increases in group 2 from 0.03% in 2014 to 0.1% in 2018 (APC, 30.57; *P* < .001). From 2010 to 2018, among patients in group 2, PBT targeted to the breast increased from 0.0% to 0.9% (APC, 51.95%), and PBT targeted to the lung increased from 0.1% to 0.7% (APC, 28.06%) (*P* < .001 for both). Use of PBT targeted to the prostate decreased from 1.4% in 2011 to 0.8% in 2014 (APC, −16.48%; *P* = .03) then increased to 1.3% in 2018 (APC, 12.45; *P* < .001). Most patients in group 1 treated with PBT had private insurance coverage in 2018 (1039 [55.4%]); Medicare was the most common insurance type among those in group 2 (1973 [52.5%]).

**Conclusions and Relevance:**

The findings of this study show an increase in the use of PBT in the US between 2004 to 2018; prostate was the only cancer site for which PBT use decreased temporarily between 2011 and 2014, increasing again between 2014 and 2018. These findings may be especially relevant for Medicare radiation oncology coverage policies.

## Key Points

**Question:**

What were the patterns of proton beam therapy (PBT) use among groups of patients with different PBT indications in the US from 2014 to 2018?

**Findings:**

In this cross-sectional study with 5 919 368 patients, PBT use increased nationally between 2004 and 2018 for both cancer sites for which PBT use is the recommended treatment modality (group 1) and for sites for which effectiveness of PBT over other radiotherapy modalities is still being investigated (group 2). Breast and prostate cancers are most frequently treated with PBT.

**Meaning:**

The findings of this study suggest that PBT uptake varies by indication group and is most commonly used to treat cancers for which PBT effectiveness is still under study.

## Teaser

This cross-sectional study assesses the changes in the use of proton beam radiotherapy in the treatment of cancers in the US over time.

## Introduction

Proton beam radiotherapy (PBT) is a form of external beam radiation used in cancer care that provides the opportunity for better precision in dose delivery than other types of external beam radiotherapy.^1^ Owing to its unique deposition characteristics, PBT is potentially superior to photon-based therapy for tumors with complex anatomy surrounded by critically sensitive tissues and for childhood cancers.^1,2^ Proton beam radiotherapy was approved for treatment of cancer in 1988 and, since then, use of PBT has increased in the US.^3^ However, evidence related to the efficacy and effectiveness of PBT varies by cancer site.

In clinical trials, PBT has demonstrated high efficacy (minimal toxic effects and local tumor control) for several rare tumors that are adjacent to critical tissues or structures and require high doses of radiation.^4,5,6,7^Proton beam radiotherapy is recommended for treatment of pediatric cancers, because minimizing late effects of radiation treatment (RT) is necessary, and in cancers where pituitary, visual, auditory, and intellectual functions might be disrupted because of RT.

High up-front capital investments and operating costs complicate the uptake of PBT.^8^ Treatment cost to payers can be double the cost of photon-based radiotherapy depending on the indication,^9,10,11,12^ and insurers may not cover treatments without clinical trial evidence to justify higher costs.^13,14^ Lack of insurance coverage is a principal barrier for enrollment in trials evaluating PBT in cancer treatment.^8,9^

The Centers for Medicare & Medicaid Services does not have a national coverage determination for PBT; instead, local coverage decisions specify conditions for payments. The first local coverage decision conditions for payment of PBT claims went into effect in 2009.^3^ Commercial insurers and state Medicaid plans have disparate definitions for medical necessity and for indications still under study and are more restrictive than Medicare in covering PBT.^8,9^

In the US, patient age and income are closely associated with health insurance coverage type. Adults aged 65 years and older are age-eligible for Medicare, and employment-based private health insurance is the main source of coverage for individuals younger than 65 years. Some individuals without access to employer-sponsored coverage are eligible for Medicaid coverage on the basis of income and other requirements determined by state policies. Other individuals can purchase health insurance coverage through the marketplace, with age informing premiums and income determining eligibility for subsidies. Therefore, age, income, and health insurance coverage type are major factors in access to PBT.

In 2014, the American Society of Radiation Oncology (ASTRO) categorized PBT clinical indications into group 1, for which health insurance coverage is recommended, and group 2, for which coverage is recommended only if additional clinical requirements are met.^15^ The ASTRO considers use of PBT reasonable in instances in which sparing the surrounding healthy tissue cannot be adequately achieved with photon-based radiotherapy and PBT use is of added clinical benefit to the patient. The guidelines were updated in 2017.^16^

Little is known about patterns of uptake of PBT according to clinical evidence used in the development of the ASTRO indications. In this study, we used national data to characterize changes in receipt of PBT by ASTRO-designated group 1 and group 2 indications as well as by patients’ age, health insurance type, and income.

## Methods

Individuals newly diagnosed with cancer between 2004 and 2018 were identified from the National Cancer Database (NCDB), a hospital-based cancer registry jointly sponsored by the American College of Surgeons and the American Cancer Society that captures approximately 72% of all cancer cases in the US from more than 1500 facilities accredited by the American College of Surgeons’ Commission on Cancer.^17^ This study followed the Strengthening the Reporting of Observational Studies in Epidemiology (STROBE) reporting guideline for cross-sectional studies and was granted exemption from review by the institutional review board of the Morehouse School of Medicine in Atlanta, Georgia, because the study was a secondary analysis of deidentified data.

The proportion of PBT facilities in operation that are included in the NCDB was determined by combining publicly available information from the Particle Therapy Co-Operative Group^18^ and the American College of Surgeons’ Commission on Cancer.^19^ To account for PBT availability, only patients diagnosed at facilities where at least 5 patients received PBT between 2004 and 2018 or who were treated by a radiation oncologist who treated at least 5 patients with PBT were included (n = 7 129 898). Patients diagnosed with a cancer site, histologic type, and stage for which no other patients in the NCDB received PBT were excluded (n = 1 211 758).

We used the ASTRO Model Policies published in 2017 to retrospectively classify patients into group 1 and group 2 according to cancer type and RT anatomic target (eTable 1 in the Supplement).^16^ Group 1 included patients treated for ocular tumors, head and neck tumors (including mouth, parotid gland, tonsil, oropharynx, nasopharynx, pyriform sinus, hypopharynx, and paranasal sinuses), central nervous system tumors (including cerebral meninges, brain, spinal cord, and other central nervous system sites), hepatocellular carcinoma, skull and spine tumors, and rhabdomyosarcoma (relevant histologic codes pooled from several different primary sites).^20^ Group 2 included patients treated for prostate, lung, breast, esophagus, pelvic (including colorectal, anal, uterine, cervical, and testicular) tumors, abdominal (including stomach, pancreas, and kidney) tumors, and thoracic lymphomas. These patients were treated while clinical evidence for medical necessity was accruing.

Self-identified race and ethnicity were ascertained from patients’ medical records. We present patient characteristics to indicate the diversity of the study population.

### Statistical Analysis

Data analysis was performed from October 4, 2021, to February 22, 2022. Patient characteristics were compared between indication groups, using χ^2^ statistics. To characterize patterns in PBT use, annual percent change (APC) was calculated by fitting a least-squares regression to the natural logarithm of PBT use rates, using diagnosis year as the independent variable. Changes in patterns (structural breaks) were identified by using the additive outliers method.^21^ Trends in PBT use through time overall, by ASTRO indication group, cancer site, age group, health insurance coverage type, and patients’ residence zip code median income quintiles were evaluated. All analyses were performed using SAS, version 9.4 (SAS Institute Inc). Statistical significance was set at a 2-sided threshold of α = .05.

## Results

Of the 5 919 368 patients eligible to receive PBT included in the study, 3 206 902 were female (54.2%) and 2 711 238 were male (45.8%) (Table). Mean (SD) age at diagnosis was 62.6 (12.3) years. Group 1 cancer sites were less common than group 2 sites. Patients diagnosed with group 2 cancer sites were more likely to be older (group 1, 58.7 [17.4] vs group 2, 63.5 [12.8] years), female (group 1, 445 063 [42.7%] vs group 2, 2 761 839 [56.6%]), reside in high-income areas (≥$69 000: group 1, 263 320 [25.5%] vs group 2, 1 394 908 [28.8%]), and have Medicare coverage (group 1, 395 628 [38.8%] vs group 2, 2 156 993 [45.0%]). Self-reported race and ethnicity for group 1 vs group 2 were Asian and Pacific Islander (39 576 [3.9%] vs 150 381 [3.1%]), Black (121 178 [11.8% vs 594 935 [12.3%]), Hispanic (77 055 [7.5%] vs 237 918 [4.9%]), White (776 497 [75.6%] vs 3 799 907 [78.8%], and other (American Indian, Aleutian, Inuit, and 2 or more races: 12 792 [1.2%] vs 41 231 [0.9%]).

**Table.** Characteristics of Patients Diagnosed With ASTRO Model Policies Group 1 and Group 2 Cancers from the National Cancer Database

Characteristic	ASTRO model policy groups, No. (%)^a^	
	Group 1	Group 2
Total	1 041 848	4 877 520
Age, y		
<15	28 781 (2.8)	5869 (0.1)
15-39	98 106 (9.4)	169 171 (3.5)
40-64	506 370 (48.6)	2 310 744 (47.4)
65-74	232 936 (22.4)	1 433 563 (29.4)
≥75	175 655 (16.9)	958 173 (19.6)
Sex		
Male	596 429 (57.2)	2 114 809 (43.4)
Female	445 063 (42.7)	2 761 839 (56.6)
Race and ethnicity^b^		
Asian and Pacific Islander	39 576 (3.9)	150 381 (3.1)
Black	121 178 (11.8)	594 935 (12.3)
Hispanic	77 055 (7.5)	237 918 (4.9)
White	776 497 (75.6)	3 799 907 (78.8)
Other	12 792 (1.2)	41 231 (0.9)
Annual income, $		
<36 000	144 747 (14.0)	599 217 (12.4)
36 000-43 999	176 210 (17.1)	756 910 (15.6)
44 000-52 999	196 890 (19.1)	884 883 (18.3)
53 000-68 999	251 559 (24.4)	1 203 140 (24.9)
≥69 000	263 320 (25.5)	1 394 908 (28.8)
Insurance		
Private	466 832 (45.8)	2 226 984 (46.5)
Uninsured	44 673 (4.4)	116 353 (2.4)
Medicaid	101 287 (9.9)	255 527 (5.3)
Medicare	395 628 (38.8)	2 156 993 (45.0)
Other	11 341 (1.1)	34 328 (0.7)
Cancer site		
Skull and spine	6262 (0.6)	0
Ocular	25 620 (2.5)	0
Rhabdomyosarcoma	4037 (0.4)	0
Head and neck	279 683 (26.8)	0
Central nervous system	537 941 (51.6)	0
Hepatocellular	188 305 (18.1)	0
Esophagus	0	94 010 (1.9)
Thoracic	0	45 985 (0.9)
Prostate	0	1 187 401 (24.3)
Lung	0	1 009 451 (20.7)
Breast	0	1 904 156 (39.0)
Pelvic	0	447 256 (9.2)
Abdominal	0	189 261 (3.9)
Diagnosis year		
2004	46 030 (4.4)	260 400 (5.3)
2005	49 118 (4.7)	267 850 (5.5)
2006	52 623 (5.1)	286 090 (5.9)
2007	56 275 (5.4)	302 072 (6.2)
2008	60 132 (5.8)	309 720 (6.3)
2009	64 407 (6.2)	317 363 (6.5)
2010	65 836 (6.3)	314 121 (6.4)
2011	69 572 (6.7)	327 577 (6.7)
2012	73 135 (7.0)	323 955 (6.6)
2013	77 396 (7.4)	336 348 (6.9)
2014	80 400 (7.7)	342 858 (7.0)
2015	84 190 (8.1)	357 845 (7.3)
2016	86 670 (8.3)	364 597 (7.5)
2017	89 455 (8.6)	380 169 (7.8)
2018	86 609 (8.3)	386 555 (7.9)

Abbreviation: ASTRO, American Society of Radiation Oncology.

^a^ All differences significant at *P* < .001.

^b^ Includes American Indian, Aleutian, Inuit, and 2 or more races. Data self-reported and given here as in the database.

**Figure 1.**
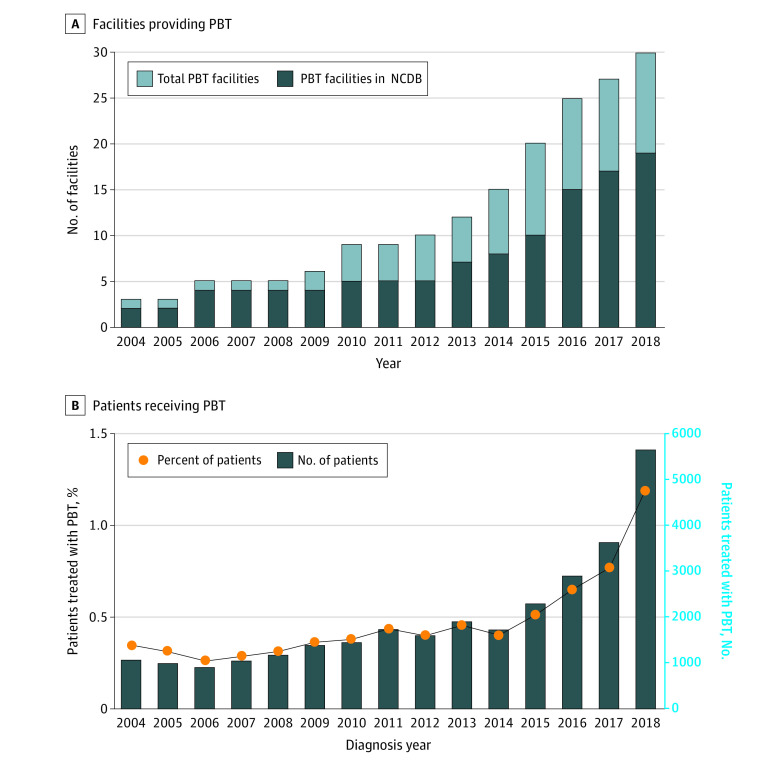
Number of Proton Beam Therapy (PBT) Facilities and PBT Use Over Time A, National number of PBT facilities identified from the Particle Therapy Co-operative Group^18^ and number of National Cancer Database (NCDB) facilities from the Commission on Cancer.^19^ B, Total number and percent of patients treated with PBT.

Of the 30 PBT facilities in clinical operation during the study period, 19 (63.3%) reported data to the NCDB. The NCDB captures RT (including PBT) that occurs outside of the reporting facility, and 14 477 patients (40.2%) treated with PBT received RT outside the reporting facility. Both the number of PBT facilities (Figure 1A)^18,19^ and use of PBT among patients in NCDB (Figure 1B) increased nationally from 0.4% in 2004 to 1.2% in 2018 (APC, 8.12%; *P* < .001).

**Figure 2.**
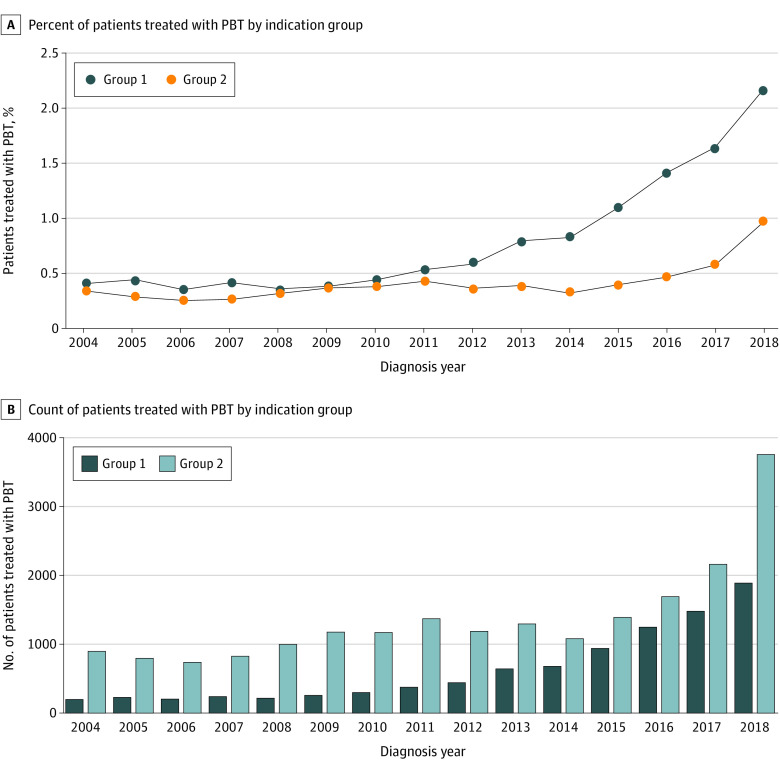
Patients Treated With Proton Beam Therapy (PBT) by American Society of Radiation Oncology Indication Groups Percent (A) and count (B) of patients receiving PBT.

Use of PBT increased significantly among patients in group 1, from 0.4% in 2010, to 2.2% in 2018 (APC, 21.97; *P* < .001), and in group 2 from 0.03% in 2014 to 0.1% in 2018 (APC, 30.57; *P* < .001) (Figure 2A). In 2018, 1876 patients (2.2%) diagnosed with group 1 cancers received PBT, compared with 3760 patients (0.9%) with group 2 cancers (Figure 2A). Most (3760 [66.7%]) patients treated with PBT in 2018 were treated for group 2 cancers (Figure 2B).

**Figure 3.**
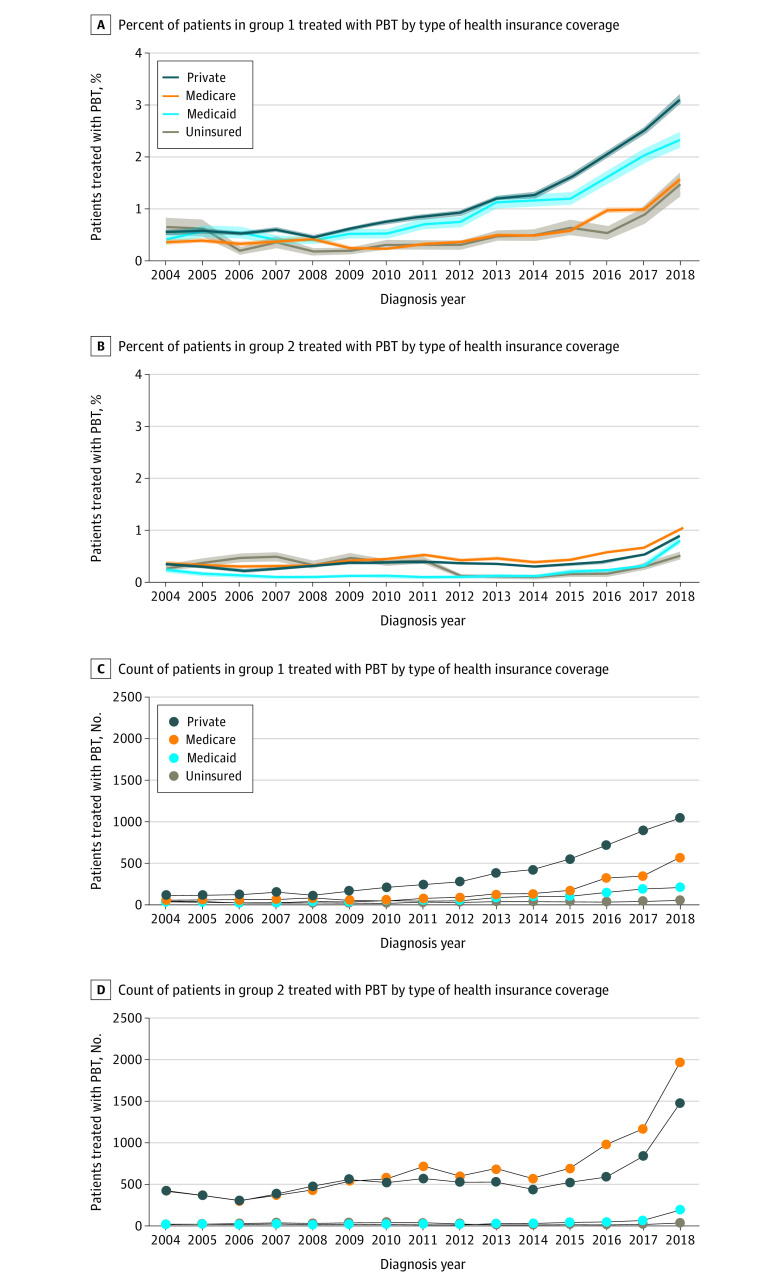
Patients Treated With Proton Beam Therapy (PBT) by American Society of Radiation Oncology Model Policy Groups and Health Insurance Coverage Proton beam therapy use by type of health insurance coverage. Percent of patients treated with PBT in group 1 (A) and group 2 (B); count of patients treated with PBT in group 1 (C) and group 2 (D).

Use of PBT for group 1 cancers increased significantly among patients with every type of insurance coverage between 2010 and 2018 (APC, 20.89 for private insurance, 22.78 for uninsured, 21.01 for Medicaid, and 28.80 for Medicare; *P* < .001 for all) (Figure 3A). In 2018, 1039 patients (3.0%) with private coverage who were diagnosed with group 1 tumors received PBT. Use of PBT in patients in group 2 increased between 2014 and 2018 among those with all types of insurance coverage (APC, 32.04 for private insurance, 28.24 for Medicare, 53.01 for Medicaid, and 51.31 for the uninsured; *P* < .001 for all) (Figure 3B). In 2018, although most patients who received PBT for group 1 cancers had private insurance (1039 of 1876 [55.4%]) (Figure 3C), Medicare was the most common coverage type among patients treated with PBT for group 2 cancers (1973 of 3760 [52.5%]) (Figure 3D).

Use of PBT for treatment of all group 1 cancer sites increased significantly between 2010 and 2018 (eTable 2 in the Supplement). Use of PBT increased most rapidly for head and neck tumors (APC, 52.0%), and central nervous system was the cancer type most frequently treated with PBT among group 1 indications in 2018 (821 patients). Among group 2 cancers, use of PBT increased between 2010 and 2018 for all cancer sites except prostate. From 2010 to 2018, among patients in group 2, PBT targeted to the breast increased from 0.0% to 0.9% (APC, 51.95%), and PBT targeted to the lung increased from 0.1% to 0.7% (APC, 28.06%) (*P* < .001 for both). Use of PBT targeted to the prostate decreased from 1.4% in 2011 to 0.8% in 2014 (APC, −16.48%; *P* = .03) then increased to 1.3% in 2018 (APC, 12.45; *P* < .001). Use of PBT increased most rapidly for breast cancer, and breast was the group 2 cancer site most frequently treated with PBT in 2018. Use of PBT for prostate cancer decreased between 2011 and 2014 and increased between 2014 and 2018 (eTable 2 in the Supplement). The decrease in PBT use for prostate cancer was not parallel with the decrease in the number of patients diagnosed with prostate cancer or treated with RT in NCDB, which started earlier, in 2008, and at a slower pace (eFigure 1 in the Supplement). Even with the significant decrease in PBT use for prostate cancer after 2011, prostate was the second most frequently treated group 2 cancer site with PBT in 2018 (eTable 2 in the Supplement).

**Figure 4.**
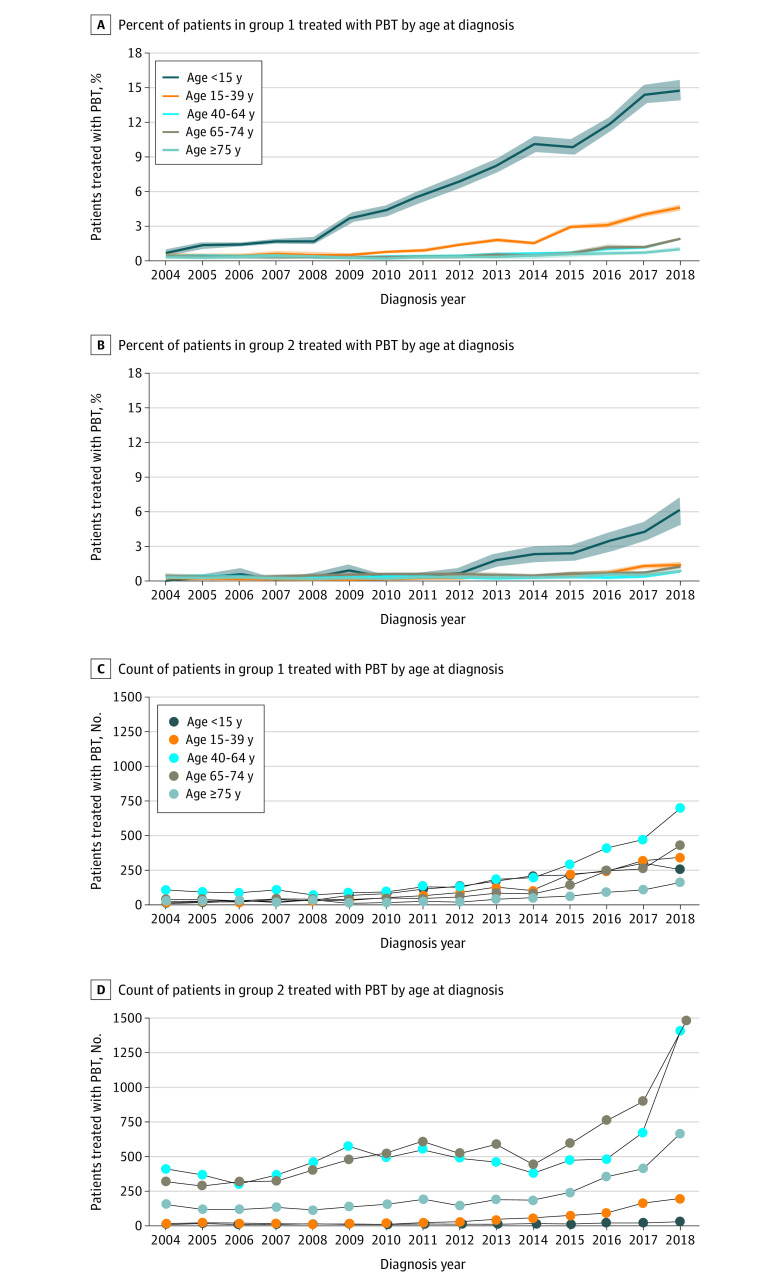
Patients Treated With Proton Beam Therapy (PBT) by American Society of Radiation Oncology Model Policy Group and Age Proton beam therapy use by age at diagnosis. Percent of patients treated with PBT in group 1 (A) and group 2 (B); count of patients treated with PBT in group 1 (C) and group 2 (D).

Use of PBT for group 1 cancers increased significantly in every age group between 2010 and 2018 for patients in group 1 and between 2014 and 2018 for those in group 2 (Figure 4). In 2018, 258 children (14.8%) diagnosed with group 1 cancers received PBT (Figure 4A). Most patients treated with PBT for group 1 indications in 2018 were diagnosed between ages 40 and 64 years (692 of 1876 [36.9%]) (Figure 4C); ages 65 to 74 years was the most common age group treated with PBT for group 2 indications in 2018 (1488 of 3760 patients [39.7%]) (Figure 4D).

Use of PBT increased significantly in every income level between 2010 and 2018 for patients in group 1 and between 2014 and 2018 for those in group 2 (eFigure 2A and 2B in the Supplement). Most patients who received PBT for treatment of both group 1 and group 2 cancers in 2018 resided in high-income areas (eFigure 2C and 2D in the Supplement).

## Discussion

In this large, comprehensive cross-sectional study, PBT use among patients newly diagnosed with cancer increased within the US between 2004 and 2018. There was a sharp increase in the number of patients treated for group 1 indications after 2010 and the number in group 2 after 2014. The increase in the total number and percent of patients treated with PBT is partly due to the increase in the number of PBT facilities in the US.^3,18^

The sharp increase in the number of patients treated with PBT targeted to anatomic sites recently included in the ASTRO Model Policies as group 1 indications could be owing to increasing adherence to mounting clinical evidence for medical necessity, even before the ASTRO guidelines were published, or owing to patients’ enrollment in the clinical trials that generated the medical evidence used to develop the Model Policies. Despite the rarity of cancer sites included in group 1 indications, more than 30% of patients treated with PBT in 2018 conformed with the ASTRO Model Policies group 1 indications.

For all group 2 indications, the ASTRO Model Policies state that additional clinical data are needed for appropriate coverage policies to be developed. In addition, patients treated under the Coverage with Evidence Development paradigm should be covered by the insurance carrier as long as the patient is enrolled in an institutional review board–approved clinical trial. However, a principal barrier for enrollment in clinical trials is health insurance coverage.^8^ Although Medicare covers all indications currently under study,^8,9^ private insurers vary greatly in their criteria for PBT coverage, even for group 1 indications, and Medicaid coverage varies by state.^22,23^

Private insurance was the most common type of coverage among patients treated with PBT for group 1 indications. Most patients treated with PBT for group 2 indications had Medicare coverage, consistent with the higher incidence of group 2 cancers in adults older than 65 years.^24^

Nearly 15% of children diagnosed with group 1 tumors were treated with PBT in 2018, and age 40 to 64 years was the most common age group treated with PBT for group 1 indications. In contrast, approximately 6% of pediatric patients and approximately 1% of patients of all other ages diagnosed with group 2 cancers were treated with PBT. Most patients with group 2 cancers treated with PBT were older adults (aged 65-74 years), who are age-eligible for Medicare coverage.

Sociodemographic differences in PBT use over time might be partly due to the most commonly diagnosed cancer types in group 1 and group 2. Cancers affecting the central nervous system, which is the cancer most frequently treated with PBT for group 1 indications, is the second most commonly diagnosed childhood cancer.^24^ Prostate, lung, and breast cancer—the nonskin cancers with the highest incidence in the US population^24^—were the most common target anatomic sites among patients receiving PBT for group 2 indications. The median age at diagnosis for prostate and lung cancer is older than 65 years.^24^ Thus, the Medicare program covers PBT for most of these patients.

The number of patients treated with PBT targeted to the prostate decreased sharply after 2011. Although the incidence of prostate cancer decreased following the 2008 and 2012 United States Preventive Services Taskforce recommendations against prostate specific antigen–based screening,^24,25^ it does not fully explain the decrease in PBT use targeted to the prostate between 2011 and 2014. Changes in health insurance coverage^26^ and publications around the time of the decrease, including a comparative effectiveness study showing that patients with prostate cancer who received PBT had a higher rate of gastrointestinal problems and did not have significantly improved outcomes compared with patients treated with intensity-modulated RT,^27^ may have contributed to the decrease. However, not all studies reported increased toxic effects with PBT.^13^

Proton beam therapy is considered reasonable in instances in which sparing the surrounding healthy tissue cannot be adequately achieved by photon-based radiotherapy and is of added clinical benefit to the patient. With the development of injectable biodegradable rectal spacers that significantly reduced radiation-induced toxic effects,^28,29^ PBT may not be of added clinical benefit to the patient with prostate cancer in terms of rectal toxic effects. To our knowledge, no study has shown a clear clinical benefit for PBT in prostate cancer, and PBT for primary treatment of prostate cancer is recommended by the ASTRO only within the context of a prospective clinical trial or registry.^13,14,16,30,31^

In contrast to those with prostate cancer, the number of patients receiving PBT targeted to the breast and lung increased significantly between 2010 and 2016, without a similar increase in incidence.^24^ Similar to prostate cancer, there is no consensus on the use of PBT for the treatment of breast^1,12,16,32,33,34,35,36^ or lung^37,38,39,40,41,42,43,44,45,46,47,48,49,50,51,52,53,54,55^ cancers. In addition, the increasing demand for PBT can be attributed, in part, to marketing by PBT facilities and patient support groups advocating for PBT.^56,57^ However, the high number of patients treated with PBT for group 2 indications does not necessarily indicate overuse of a therapy with unproven benefits. It is possible that a large proportion of patients receiving PBT are enrolled in clinical trials or registry studies aimed at evidence development.

For adults younger than 65 years, who are not age-eligible for Medicare, private insurance approval can be a barrier for enrollment in clinical trials necessary to develop evidence-based coverage policies.^8,9,22,23^ Moreover, in July 2019, with the goal of reducing Medicare spending and improving quality of care, the Centers for Medicare & Medicaid Services proposed to test an episode-based payment model for radiation oncology, citing evidence of overuse of expensive new therapies.^58^ Continuous monitoring of how insurance coverage policies affect both PBT use and enrollment in clinical trials that generate medical evidence for the role of PBT in treating group 2 cancers will be vital.^9^

### Limitations

This study has limitations. These limitations include the lack of information on some qualifying characteristics listed by the ASTRO Model Policies for PBT treatment, especially among group 2 indications; lack of information about treatment recommendations and the decision-making process; clinical trial enrollment or relevant outcomes of PBT, including toxic effects and lasting effects of treatment; and lower prostate cancer capture in the NCDB (58% of patients with prostate cancer are captured in the NCDB compared with 72% of all patients with newly diagnosed cancer).^17^ As of 2018, 66% of PBT facilities in the US reported to the NCDB, and the NCDB captures RT, including PBT, received at facilities other than the reporting facility.^59^ Therefore, it is likely that changes in PBT captured in the NCDB are representative of national patterns. However, for patients treated outside of NCDB facilities, information about treating facility type is unavailable. Therefore, evaluation of the patterns of PBT uptake by facility type, volume, or distance was not possible. Because the NCDB includes information on first-course treatment for incident cancers only, data on use of PBT for recurrent disease or reirradiation are not available. Monitoring PBT use will be important for future research. Nonetheless, the NCDB implements stringent data quality, standardization, and ascertainment methods, and patients included in the NCDB are similar to patients included in population-based databases.^17^

## Conclusions

This study provides useful information about national patterns in uptake of PBT by ASTRO indications by health insurance coverage and patient characteristics. The number of patients receiving PBT increased between 2004 and 2018, including a larger proportion of patients being treated for group 1 indications. Despite the variability in criteria for PBT coverage among insurance providers, the number of patients with private insurance who are treated with PBT for group 1 indications has increased, especially among pediatric patients. Adoption of the ASTRO Model Policies by private and public insurers could facilitate access to patients for whom evidence suggests PBT is superior to photon-based RT. Furthermore, adoption of the policies could help resolve lack of evidence for medical necessity for group 2 indications by requiring that patients treated for group 2 indications, who are most frequently insured by Medicare, be enrolled in clinical trials.

## Notes

Supplement. **eTable 1.** List of Topography Codes for the American Society for Radiation Oncology Group 1 Proton Beam Therapy Indication Cancer Types Based on the *International Classification of Diseases for Oncology* (*Third Edition*, *ICD-O-3*) **eTable 2.** Trends in Use of PBT by ASTRO Model Policy Group 1 and Group 2 Cancer Sites, (NCDB 2004-2018) **eFigure 1.** Number of Patients With Prostate Cancer Diagnosed With Prostate Cancer and Treated With Radiation Therapy Targeted to the Prostate, NCDB (2004-2018) **eFigure 2.** Patients Treated With PBT (Percent and Count) by ASTRO Model Policy Groups and Median Zip Code Income Level Quintiles, NCDB (2004-2018)
