# Supplementary material for: Assessment of Proton Beam Therapy Use Among Patients With Newly Diagnosed Cancer in the US, 2004-2018
Source: JAMA Netw Open. 2022 Apr 27;5(4):e229025. doi: 10.1001/jamanetworkopen.2022.9025 (PMC9047654; doi:10.1001/jamanetworkopen.2022.9025)
Supplement: Supplement. — eTable 1. List of Topography Codes for the American Society for Radiation Oncology Group 1 Proton Beam Therapy Indication Cancer Types Based on the International Classification of Diseases for Oncology (Third Edition, ICD-O-3) eTable 2. Trends in Use of PBT by ASTRO Model Policy Group 1 and Group 2 Cancer Sites, (NCDB 2004-2018) eFigure 1. Number of Patients With Prostate Cancer Diagnosed With Prostate Cancer and Treated With Radiation Therapy Targeted to the Prostate, NCDB (2004-2018) eFigure 2. Patients Treated With PBT (Percent and Count) by ASTRO Model Policy Groups and Median Zip Code Income Level Quintiles, NCDB (2004-2018) [file jamanetwopen-e229025-s001.pdf]

Supplementary Online Content

Nogueira LM, Jemal A, Yabroff KR, Efstathiou JA. Assessment of proton beam therapy use among patients with newly diagnosed cancer in the US, 2004-2018. *JAMA Netw Open*. 2022;5(4):e229025. doi:10.1001/jamanetworkopen.2022.9025

eTable 1. List of Topography Codes for the American Society for Radiation Oncology Group 1 Proton Beam Therapy Indication Cancer Types Based on the *International Classification of Diseases for Oncology* (*Third Edition*, *ICD-O-3*)
eTable 2. Trends in Use of PBT by ASTRO Model Policy Group 1 and Group 2 Cancer Sites, (NCDB 2004-2018)
eFigure 1. Number of Patients With Prostate Cancer Diagnosed With Prostate Cancer and Treated With Radiation Therapy Targeted to the Prostate, NCDB (2004-2018)
eFigure 2. Patients Treated With PBT (Percent and Count) by ASTRO Model Policy Groups and Median Zip Code Income Level Quintiles, NCDB (2004-2018)

This supplementary material has been provided by the authors to give readers additional information about their work.

© 2022 Nogueira LM et al. *JAMA Network Open.*

eTable 1. List of topography codes for the American Society for Radiation Oncology Group 1 proton beam therapy indication cancer types based on the *International Classification of Diseases for Oncology* (*Third Edition*, *ICD-O-3*)

ASTRO Indication Group	ICD-O-3 topology	ICD-O-3 Histology
**Group 1**		
**Head & Neck**		
Oral Cavity and Pharynx	C100-C109, C129, C130-C139, C140, C142, C148	8010, 8020, 8041, 8070-8072, 8082, 8083, 8140, 8147, 8200, 8430, 8500, 8502, 8550, 8562, 8940, 8941, 8982, 9370
Larynx	C320-C329	8070, 8071
Nose, Nasal Cavity and Middle Ear	C300-C301, C310-C319	8010, 8013, 8020, 8032, 8070-8072, 8074, 8083, 8140, 8144, 8200, 8246, 8720, 8746, 8800, 8801, 8890, 9220, 9370, 9500, 9522
**CNS**		
Brain	C710-C719	8000, 8800, 8963, 9064, 9065, 9070, 9085, 9150, 9231, 9240, 9350, 9351, 9370, 9380-9382, 9390-9393, 9400, 9401, 9411, 9420, 9421, 9424, 9440-9442, 9450, 9451, 9470, 9471, 9473, 9474, 9505, 9506, 9508, 9560
Cranial Nerves, Other Nervous System	C710-C729	8815, 9064, 9150, 9370, 9380, 9394, 9400, 9421, 9530-9532, 9534, 9537-9539, 9560
Other Intracranial, Intraspinal Neoplasm	C751-C753	8272, 9064, 9080, 9085, 9350-9352, 9361, 9362, 9370, 9380
**Hepatocellular**	C220-C221	8140, 8160, 8170, 8171, 8174, 8180
**Skull and Spine**	C410-C412, C414	9180, 9181, 9220, 9231, 9240, 9260, 9370-9372,
**Ocular**	C690-C699	8070, 8071, 8140, 8200, 8720, 8730, 8770-8772, 8774, 8800, 8858, 9510
**Rhabdomyosarcoma**	Any site	8900, 8902, 8910, 8912, 8920, 8991
**Group 2**		
**Prostate**	C619	8040, 8140, 8480, 8500, 8550
**Lung**	C8340-C8349	8000, 8010, 8012, 8013, 8041, 8045, 8046, 8070-8072, 8083, 8140, 8200, 8230, 8240, 8246, 8250, 8253, 8255, 8260, 8480, 8481, 8550, 8551, 8560, 8574
**Breast**	C500-C509	8010, 8050, 8140, 8201, 8211, 8230, 8255, 8480, 8490, 8500, 8501, 8503, 8504, 8507, 8510, 8520, 8522, 8523, 8524, 8530, 8541, 8575
**Pelvic**		
Colorectal	C187, C199, C209	8140, 8083, 8210, 8261, 8263, 8480, 8481, 8490
Anal	C210, C211	8070-8072, 8083, 8124, 8140
Uterus	C541, C559	8140, 8323, 8441, 8460, 8570, 8950, 8980

© 2022 Nogueira LM et al. *JAMA Network Open.*

Cervix	C530-C539	8070-8072, 8140, 8560
Testis	C621, C629	9061
**Abdominal**		
Pancreas	C250-C259	8000, 8010, 8140, 8480, 8500, 8550, 8560
Stomach	C160, C162, C163, C169	8010, 8070, 8140, 8144, 8490
Kidney	C649	8690
**Esophagus**	C150-C159	8010, 8070, 8140, 8244, 8246, 8255, 8480, 8490, 8560, 8574
**Thoracic**		
Non-Hodgkin Lymphoma	C379, C381, C383	9679
Hodgkin Lymphoma	C770, C771, C773, C778, C779	9650, 9652, 9659, 9663, 9664

© 2022 Nogueira LM et al. *JAMA Network Open.*

eTable 2. Trends in use of PBT by ASTRO Model Policy Group 1 and Group 2 cancer sites, (NCDB 2004-2018)

	**Diagnosis Year**									
	**004**	**010**	**011**	**012**	**013**	**014**	**015**	**016**	**017**	**018**
**C ancer Site**	**(%)**	**(%)**	**(%)**	**(%)**	**(%)**	**(%)**	**(%)**	**(%)**	**(%)**	**(%)**
Gr oup 1										
Head & Neck	0 (0.3)	7 (0.1)	3 (0.2)	5 (0.2)	5 (0.5)	02 (0.5)	01 (0.9)	25 (1.4)	38 (1.9)	14 (3.0)
CNS	5 (0.2)	15 (0.3)	52 (0.4)	94 (0.5)	64 (0.7)	05 (0.7)	08 (1.0)	40 (1.2)	49 (1.4)	21 (1.9)
Hepatocell ular	SU (0.1)	SU (0.1)	SU (0.1)	SU (0.1)	7 (0.1)	6 (0.2)	8 (0.2)	0 (0.3)	2 (0.3)	3 (0.4)
Skull and Spine	9 (6.3)	1 (8.4)	3 (10.7)	7 (11.7)	7 (11.9)	5 (9.4)	4 (16.4)	9 (14.7)	13 (20.4)	3 (16.1)
Ocular	3 (5.7)	2 (6.0)	01 (6.2)	6 (5.7)	23 (6.8)	18 (6.6)	33 (6.5)	69 (8.3)	38 (6.7)	24 (6.7)
Rhabdom yosarcoma	SU (0.5)	3 (8.2)	6 (10.4)	2 (11.2)	5 (18.7)	1 (21.1)	7 (21.0)	1 (20.7)	4 (25.5)	1 (22.8)
Gr oup 2										
Prostate	83 (0.8)	048 (1.3)	200 (1.4)	05 (1.3)	32 (1.2)	49 (0.8)	06 (0.8)	57 (1.0)	77 (1.0)	097 (1.3)
Lung	1 (0.1)	0 (0.1)	6 (0.1)	9 (0.1)	24 (0.2)	35 (0.2)	12 (0.3)	97 (0.3)	73 (0.3)	38 (0.7)
Breast	94 (0.2)	7 (0.0)	6 (0.0)	7 (0.1)	68 (0.1)	34 (0.2)	42 (0.2)	51 (0.3)	55 (0.4)	458 (0.9)
Pelvic	9 (0.1)	SU (0.0)	SU (0.0)	SU (0.0)	0 (0.1)	3 (0.1)	6 (0.1)	6 (0.2)	3 (0.2)	99 (0.8)
Abdominal	SU (0.1)	7 (0.1)	9 (0.1)	3 (0.2)	7 (0.4)	0 (0.3)	7 (0.4)	3 (0.4)	3 (0.4)	01 (0.6)
Esophagu s	SU (0.1)	SU (0.1)	SU (0.1)	9 (0.3)	6 (0.5)	5 (0.4)	0 (1.0)	4 (1.3)	26 (1.7)	89 (2.3)
Thoracic	SU (0.4)	SU (0.2)	SU (0.5)	7 (0.5)	2 (1.0)	8 (1.1)	7 (1.3)	3 (1.8)	3 (2.0)	8 (2.2)

Notes: DSU: Data suppressed due to confidentiality, APC: Annual Percent Change
Counts and percent from 2005-2009 (no significant APC) suppressed for clarity.
[a] Between 2010 and 2018, [b] between 2011 and 2014, [c] between 2014 and 2018

© 2022 Nogueira LM et al. *JAMA Network Open.*

eFigure 1. Number of patients with prostate cancer diagnosed with prostate cancer and treated with radiation therapy targeted to the prostate, NCDB (2004-2018).

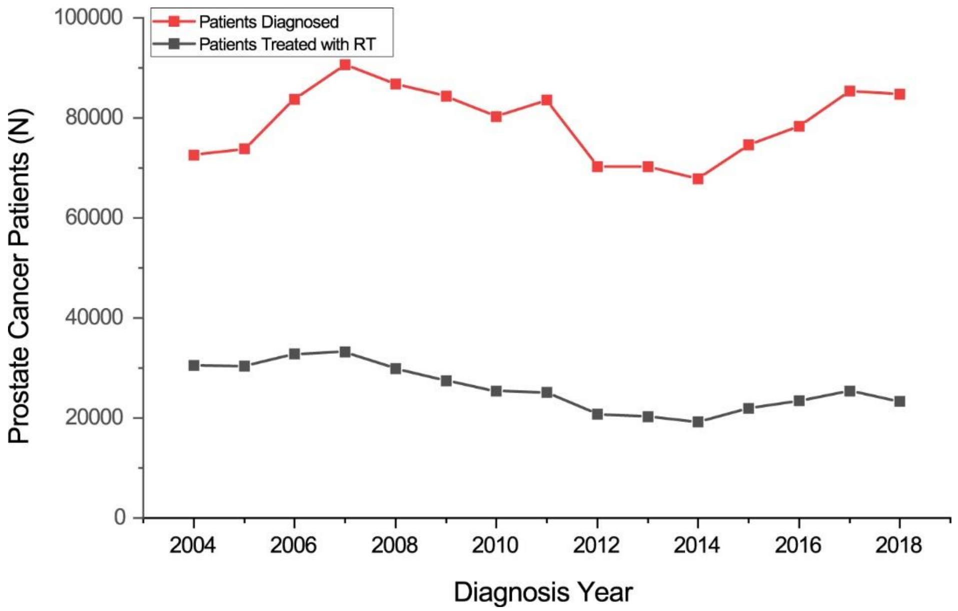

© 2022 Nogueira LM et al. *JAMA Network Open.*

eFigure 2. Patients treated with PBT (percent and count) by ASTRO Model Policy Groups and median zip code income level quintiles, NCDB (2004-2018)

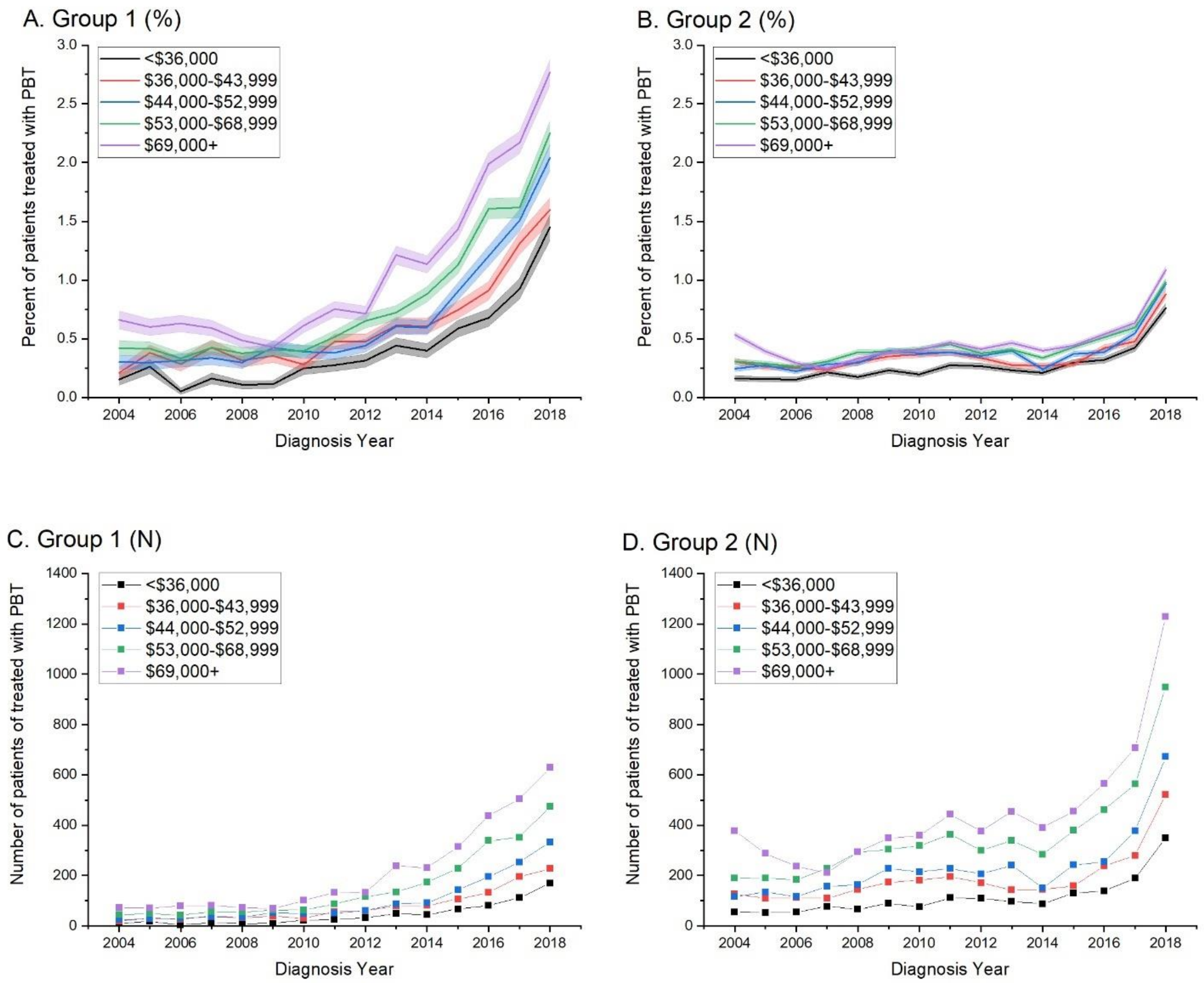

© 2022 Nogueira LM et al. *JAMA Network Open.*
